# Effect of Polymerization Time and Home Bleaching Agent on the Microhardness and Surface Roughness of Bulk-Fill Composites: A Scanning Electron Microscopy Study

**DOI:** 10.1155/2019/2307305

**Published:** 2019-06-02

**Authors:** Zümrüt Ceren Özduman, Magrur Kazak, Mehmet Ali Fildisi, Rümeysa Hatice Özlen, Evrim Dalkilic, Nazmiye Donmez

**Affiliations:** ^1^Department of Restorative Dentistry, Faculty of Dentistry, Bezmialem Vakif University, 34093 Istanbul, Turkey; ^2^Bahcesehir University, School of Dental Medicine, Restorative Dentistry Department, Istanbul, Turkey

## Abstract

**Objective:**

The aim of this study is to evaluate the microhardness and surface roughness of two different bulk-fill composites polymerized with light-curing unit (LCU) with different polymerization times before and after the application of a home bleaching agent.

**Materials-Methods:**

For both microhardness and surface roughness tests, 6 groups were prepared with bulk-fill materials (SonicFill, Filtek Bulk Fill) according to different polymerization times (10, 20, and 30 s). 102 specimens were prepared using Teflon molds (4 mm depth and 5 mm diameter) and polymerized with LCU. 30 specimens (*n* = 5) were assessed for microhardness. Before home bleaching agent application, the bottom/top (B/T) microhardness ratio was evaluated. After bleaching agent application, the microhardness measurements were performed on top surfaces. Roughness measurements were performed in 72 specimens (*n* = 12) before and after bleaching application. Additionally, for SEM analyses, two specimens from all tested groups were prepared before and after bleaching agent application. The data B/T microhardness ratio before bleaching was analyzed by two-way ANOVA and Tukey's HSD test. The data from the top surface of specimens' microhardness before and after bleaching were analyzed using Wilcoxon signed-rank test, Kruskal-Wallis, Mann-Whitney *U* tests. The data from surface roughness tests were statistically analyzed by multivariate analysis of variance and Bonferroni test (*p* < 0.05).

**Results:**

The B/T microhardness ratio results revealed no significant differences between groups (*p* > 0.05). Comparing the microhardness values of the composites' top surfaces before and after bleaching, a significant decrease was observed exclusively in FB30s (*p* < 0.05). No significant differences in surface roughness values were observed when the groups were compared based on bulk-fill materials (*p* > 0.05) while the polymerization time affected the surface roughness of the SF20s and SF30s groups (*p* < 0.05). After bleaching, surface roughness values were significantly increased in the SF20s and SF30s groups (*p* < 0.05).

**Conclusion:**

The clinicians should adhere to the polymerization time recommended by the manufacturer to ensure the durability of the composite material in the oral environment.

## 1. Introduction

Resin-based composite (RBC) is one of the most commonly used materials in restorative dentistry. However, RBC composition has significantly improved, leading to better esthetics, mechanical properties, and clinical durability. In recent years, manufacturers have introduced bulk-fill resin-based composite materials that can fill cavities up to 4–6 mm at once [1]. However, optimum polymerization to the full depth of the RBC is very important to obtain proper mechanical and physical properties.

Polymerization of the restoration is directly related to the material's organic and inorganic composition as well as the type and morphology of the filler contents [2]. Furthermore, polymerization is influenced by the polymerization time, light spectrum, and distance of the restoration to the light-curing unit [3–5]. Hardness measurement has been shown to be a practical and indirect method determining the material's degree of polymerization. Bottom/top surfaces' hardness profiles can be used to alternatively measure the depth of cure. [6]

LED light-curing units (LCUs) have advantages, such as shorter exposure times and longer service time, compared to halogen LCUs [7]. Recently, numerous advancements in LED-LCUs have occurred. First- and second-generation LED-LCUs have a narrow monowave light spectrum (450-470 nm) that can polymerize 2 mm thick resin samples in 20-40 s. However, some manufacturers of bulk-fill composite and other dental resin materials have started to use alternative photoinitiators to increase polymerization. There is not enough research in the literature regarding the effect of extended or decreased light-curing duration on the mechanical performance of new bulk-fill composites. There are contradictory results in the literature on the light-curing duration of RBCs [4, 8]. It is important to optimize the curing efficiency to reduce chair time and prevent thermal damage of light-curing units. However, extending the light-curing time is commonly considered to impact the number of photons delivered to the RBC, which may increase the physical properties of the material [9].

Bleaching procedure on discolored teeth is a relatively conservative esthetic procedure. Therefore, the public demand for whiter teeth has increased in recent years. Nightguard home bleaching uses a relatively low level of whitening agent and is applied to the teeth via a tray that is worn nightly for the duration of at least two weeks [10]. Bleaching materials and techniques may differently influence RBCs [11, 12]. Application of the bleaching agent to RBCs may result in changes in microhardness and roughness, especially when treatment is not performed under a dentist's supervision [13, 14]. Any change in hardness from bleaching may affect the clinical durability of restorations given that hardness is related to the materials' strength [15]. An increase in roughness results in the accumulation of plaque and the formation of biofilms, which are the main causes of caries and periodontal diseases [16]. To the authors' knowledge, no study in the literature has investigated the effect of bleaching agent on the microhardness and surface roughness of bulk-fill composites with different polymerization times.

The first aim of this study is to evaluate the effect of different polymerization times on the microhardness and roughness properties of novel bulk-fill composites. The second aim of this study is to evaluate the effect of home bleaching agent on the microhardness and surface roughness of two bulk-fill composites with different polymerization times. The null hypothesis is that polymerization time affects the microhardness and surface roughness of bleached and unbleached bulk-fill composites.

## 2. Materials and Methods

In the present study, two different bulk-fill composite resin materials were used (SonicFill (Kerr Corporation Orange, CA, USA) and Filtek Bulk Fill (3M ESPE, St. Paul, MN, USA)) (Table 1). For both Vickers microhardness and surface roughness tests, 6 groups were prepared with bulk-fill materials according to the different polymerization times (10 seconds (s), 20 s, and 30 s) as follows:

- Group FB 10 s: Filtek Bulk Fill polymerized for 10 s.
- Group FB 20 s: Filtek Bulk Fill polymerized for 20 s.
- Group FB 30 s: Filtek Bulk Fill polymerized for 30 s.
- Group SF 10 s: SonicFill polymerized for 10 s.
- Group SF 20 s: SonicFill polymerized for 20 s.
- Group SF 30 s: SonicFill polymerized for 30 s.

A polywave third-generation high-intensity LED-LCU (Valo, Ultradent, South Jordan, USA) (wavelength 395-480 nm) was used to polymerize the bulk-fill materials.

### 2.1. Microhardness Measurement

Thirty cylindrical specimens (*n* = 5) for 6 tested groups (4 mm in depth and 5 mm in diameter) were prepared using Teflon molds. The specimens were covered on both sides with a mylar strip and a thin rigid microscope slide to obtain a flat polymerized surface. Photoactivation was performed by positioning the light-guide tip such that it was in contact with the glass slide on the top surface of the specimen. The specimens were removed from the mold and stored at 100% humidity and 37°C for 24 hours. Vickers microhardness measurements were made using an HMV Microhardness Tester (Shimadzu, Japan) at a load of 200 g for 10 s on the top and bottom surfaces. Three indentations were made on the top (upper) and bottom (lower) surfaces of each specimen, and the Vickers Hardness Number (VHN) of each surface was recorded as the average of these readings. The microhardness measurements were repeated after home bleaching regimens performed for 1 hour × 14 days with 16% carbamide peroxide (CP) agent (Bite and White (Cavex, Netherland)). The bleaching agent was applied on one surface of the sample using a microbrush and left for 1 hour according to the manufacturers' instructions. After each bleaching procedure, the samples were washed with a water jet spray for 60 s. Then, the samples were stored in artificial saliva until the bleaching procedures were performed on the following day.

### 2.2. Roughness Measurements

In total, 72 composite discs (*n* = 12) for 6 tested groups were prepared by following the procedures mentioned above. Top surfaces of the specimens were polished with polishing disks (Sof-Lex, 3M ESPE, USA) under water cooling. The specimens were stored in 100% humidity at 37°C for 24 hours. Then, the surface roughness was measured using a profilometer (MarSurf M 300 C; Mahr GmbH, Göttingen, Germany) with a tracing length of 5.6 mm and a cutoff value of 0.8 mm. A reading was obtained using a diamond stylus moved at 0.5 mm/s, and arithmetic roughness (Ra) was recorded. This procedure was repeated for four positions (in each quadrant in a clockwise direction) on the same specimen, and the arithmetic mean was obtained from these values. The mean surface roughness values were calculated for each specimen [17]. The roughness measurements were repeated after home bleaching regimens performed for 1 hour × 14 days. Before and after bleaching agent application, one specimen from all the tested groups was prepared for the SEM (*Zeiss EVO LS 10*, Carl Zeiss NTS, Germany). Composite surfaces were sputter coated with gold and palladium alloy in a vacuum evaporator (Emitech *SC7620 Sputter Coater*; *Quorum* Technologies, UK), and photomicrographs of representative areas were obtained at ×1000 magnifications.

### 2.3. Statistical Analyses

The data of bottom/top (B/T) ratios of VHN were tested for normal distribution using the Kolmogorov–Smirnov and Shapiro–Wilk tests. Since the data (N) were normally distributed, parametric analyses method was used. The data were analyzed using the IBM Statistical Package for Social Sciences (SPSS) 22 Software for Windows. Within-group differences were analyzed by two-way analysis of variance (ANOVA), and Tukey's honest significant difference (HSD) tests were used at a significance level of 0.05.

Also, the data from the top surface of the specimens' microhardness before and after bleaching were tested for normal distribution using the Kolmogorov–Smirnov and Shapiro–Wilk tests. Since the data (N) were nonnormally distributed, nonparametric analyses method was used. The data were analyzed using the IBM Statistical Package for Social Sciences (SPSS) 22 Software for Windows. Differences between pre- and postbleaching results were analyzed by Wilcoxon signed-rank test. Additionally, percentage alterations of bleaching procedure were determined (pre-post differences) by using Kruskal-Wallis and Mann-Whitney *U* tests at a significance level of 0.05.

The data from before and after bleaching surface roughness were tested for normal distribution using the Kolmogorov–Smirnov and Shapiro–Wilk tests. Since the data (N) were normally distributed, parametric analyses method was used. Data were subjected to analysis of variance for repeated measures. Multiple comparisons were made using the Bonferroni test at a significant level of 0.05. Percentage alterations of bleaching procedure (before-after differences) were analyzed with Kruskal-Wallis and Mann-Whitney *U* tests. The significance level was set at 0.05.

## 3. Results

### 3.1. Microhardness

B/T microhardness ratio of the groups before bleaching is presented in Table 2. No significant differences were noted between the groups before bleaching (*p* > 0.05). Microhardness measurements of the top surfaces of the composites before and after bleaching are presented in Table 3. When the microhardness measurements of the top surfaces of the composites before and after bleaching were compared, a significant decrease was exclusively observed in the FB 30 s group (*p* < 0.05) (Table 3). Percentage alteration of the microhardness values of composites (between before and after bleaching measurements) and *p* values is presented in Table 4. No significant difference was determined between the percentage alteration of the microhardness values of the tested groups.

### 3.2. Surface Roughness

Surface roughness measurements of the top surfaces of all groups before and after bleaching are presented in Table 5. In the measurements of surface roughness before bleaching, no significant differences were determined between the FB groups (*p* > 0.05). However, there was a significant difference between the SF20 and SF30 groups (*p* < 0.05). When the surface roughness of the top surfaces of the composites before and after bleaching was compared, no significant differences were noted among the FB 10 s, FB 20 s, FB 30 s, and SF 10 s groups (*p* > 0.05). After bleaching, the surface roughness values were significantly increased in the SF 20 s and SF 30 s groups (*p* < 0.05). Percentage alteration of the roughness values of composites (between before and after bleaching measurements) and *p* values are presented in Table 6. Only in the SF groups, a significant difference was determined between the percentage alteration of the roughness values of SF 10 s and SF 30 s.

### 3.3. SEM Examination

**Figure 1:**
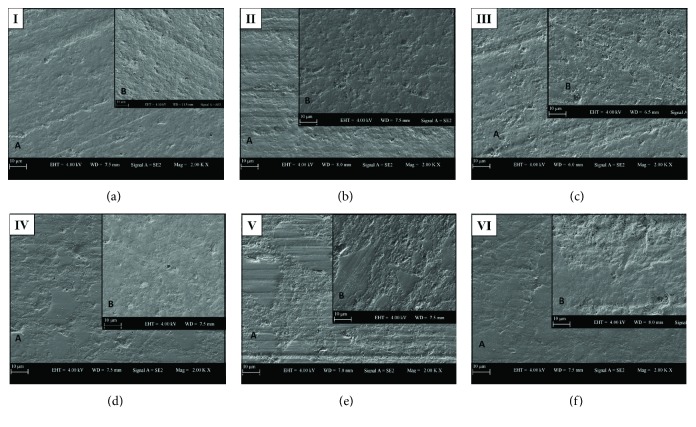
The SEM images of two bulk-fill composite groups (polymerized for 10 s, 20 s, and 30 s) before (A) and after (B) bleaching ((a) group FB 10 s; (b) group FB 20 s; (c) group FB 30 s; (d) group SF 10 s; (e) group SF 20 s; (f) group SF 30 s).

The SEM images of the two bulk-fill composite groups (polymerized for 10 s, 20 s, and 30 s) before and after bleaching are presented in Figures 1(a)–1(f).

SEM images confirmed the results of the surface roughness test in this study. Slight surface changes were determined after bleaching procedures, such as less uniform surfaces with resin removal, dislodged particles, and protruding filler particles in all bulk-fill groups (Figure 1). SEM images of the SF 20 s and SF 30 s groups revealed that surface roughness increased after bleaching more dramatically (Figures 1(e) and 1(f)).

## 4. Discussion

In the present study, the effect of polymerization time and home bleaching agent on the microhardness and surface roughness of two bulk-fill composites was determined. The results showed that before bleaching, the B/T microhardness ratio of different bulk-fill materials did not differ depending on the polymerization time. But surface roughness of SonicFill bulk-fill composite differed depending on the polymerization time. In addition to that, home bleaching agent affected the microhardness and surface roughness of different bulk-fill materials with different polimerization times. Thus, the null hypothesis was partially rejected.

Surface hardness is one of the most important mechanical properties of composite materials. Sufficient surface hardness of the composite restoration is necessary, especially in posterior stress-bearing areas. In the literature, most of the hardness measurements are performed using the Vickers microhardness test [18, 19]. In this study, the depth of cure was measured using the microhardness test method that is also practical to determine the degree of conversion [20]. The chemical structure and composition of the dimethacrylate monomer and the type of light-curing device represent some of the factors that can affect the surface hardness of composite material [21, 22]. In the present study, a polywave third-generation high-intensity LED-LCU was used to polymerize both bulk-fill materials. Regarding the composition of the material, inorganic filler particle and hardness values are positively correlated given that a decrease in filler content results in reduced mean hardness values [23, 24]. In the present study, no significant difference was determined between the B/T microhardness ratio of the bulk-fill materials polymerized with LED-LCU. This finding may be due to the similar filler weights of the materials (Filtek Bulk Fill: 76.5%, SonicFill: 83.5%).

The polymerization time is another important factor affecting the curing of resin composites. In the current study, three different polymerization times were also evaluated. Borges et al. [25] evaluated the microhardness of eight low-viscosity composite materials on top surfaces polymerized with one LED-LCU (1264 mW/cm^2^) at five polymerization times (10, 20, 30, 40, 50, and 60 s). They concluded that polymerization time did not affect the microhardness results of the top surfaces of low-viscosity composite materials. In contrast to these results, Lima et al. [26] evaluated the microhardness of nanofilled composite material on top and bottom surfaces with LED-LCU (800 mW/cm^2^) at two different polymerization times (20 s or 40 s). These researchers suggest that the polymerization time increased the microhardness of the nanofilled composites at the top and bottom surfaces. The different results between these studies may be attributed to the different power intensities of the LED-LCUs used. Although lower power intensity with an enhanced curing time seems to provide an advantage on the microhardness of the composite material, higher power intensity did not change the results. In the current study, high power intensity LED-LCUs with three (10, 20, and 30 s) different polymerization times were used. The results of our study demonstrated that different polymerization times did not result in any differences in the B/T microhardness ratio of the bulk-fill composites.

In previous studies, different results were reported about the effect of the home bleaching agents on the microhardness of RBC. Halvorson et al. revealed a significant decrease in the hardness of the composite resins after bleaching treatment (10% CP for 14 days) when the process was performed at body temperature (37°C), while the surface microhardness remained unchanged at room temperature (25°C) [27]. In another study, when 20% concentrated CP gels were applied on the composite and stored at 37°C during the bleaching period, microhardness increased [28]. Hannig et al. reported a significant decrease in the microhardness of bleached composite resins when 10% CP bleaching agent was used at 37°C [11]. In a study by Yu et al., the change in the surface microhardness of the bleached (10% CP) composite was evaluated at different environmental temperatures (25°C and 37°C), and the difference was not statistically significant [29]. Results from the current study indicate that 16% carbamide peroxide at room temperature did not affect the microhardness of FB polymerized for 20 s and SF for 10 s, which are the conditions recommended by the manufacturers. The discrepancy between our study and previous studies might be related to the differences between the temperatures and bleaching agents used and restorative materials tested.

In the present study, the effects of home bleaching agent on the microhardness of bulk-fill composites with extended polymerization times were evaluated. Home bleaching agent did not change the microhardness results in the SF groups polymerized for extended times (20 s, 30 s), while a significant decrease was determined in the FB groups polymerized for an extended time (30 s). Although there seems to be a decline, the microhardness value of the composites after bleaching agent for FB 30 s was similar to that noted in other FB groups.

In addition to surface hardness, the surface roughness is another important factor affecting the clinical success of resin composite restorations. The longevity and aesthetic appearance of the composite restorations are dependent on the quality of the polishing and surface roughness. One of the factors affecting the properties of RBC is the filler size. The type, shape, and amount of filler particles are other factors that may influence the performance of dental composites [30]. In the present study, all the groups were polished sequentially from coarse to fine with aluminum oxide abrasive discs. When the surface roughnesses of bulk-fill materials before bleaching procedures were compared with each other, no significant differences were observed. This finding could be related to the particle sizes of the filler. Filtek Bulk Fill has an average particle size ranging from 4-100 nm (4 to 11 nm zirconia particles, 20 nm silica, and 100 nm ytterbium trifluoride), while the manufacturer did not report the particle size of SonicFill bulk fill. However, both bulk-fill materials contain nanosize filler particles that may cause similar roughness values obtained in this study.

The results of this in vitro study demonstrated that the surface roughness values of all groups increased after bleaching treatment. However, the change was not statistically significant when the bulk-fill composites were polymerized according to the manufacturers' recommended curing time (10 s for SF and 20 s for FB). Although the tested composite materials in the literature were not bulk-fill materials, Polydorou et al. found that 15% CP treatment caused no significant alteration on hybrid, flowable, microhybrid, and nanohybrid composite resins, which is similar to our study [31].

The effects of bleaching gels are related to the oxidation process, which mainly influences the organic matrix of the composite resin. The organic matrix facilitates water absorption and leads to particle loss, thereby increasing roughness [14, 32]. In contrast, the inorganic content of composite resin exhibits resistance to bleaching [33, 34]. Furthermore, it is assumed that different materials respond differently after whitening procedures [35]. To the authors' knowledge, no study has investigated the influence of home bleaching agents on the surface roughness of bulk-fill composites polymerized at different times. In the present study, extended light curing (20 s, 30 s) applied to the SF groups caused statistically significant increases on surface roughness after the bleaching agent was used. Thus, any difference in surface roughness is expected to occur in resin content. In the literature, the increase of surface roughness is attributed to the degradation of the resin matrix and the subsequent debonding of the resin-filler interfaces, which would lead to dislodgment and elution of fillers [31, 36]. In our study, an extended polymerization time could explain the changes in the resin matrix of SF, rendering it prone to degradation.

Within the limitations of this study, the current results were achieved in a laboratory under specific conditions with direct contact with the resin composite specimens (no distance between the LCU and the surface), which is generally impossible to attain clinically in the mouth. It is difficult to achieve perfect working conditions in clinical practice. Therefore, less microhardness with increased roughness can be expected. However, in the present study, limited sample size for microhardness evaluation may not reflect the anticipated results. Detailed further studies are needed to clarify the microhardness alterations in bleached composite surfaces.

## 5. Conclusion

Some clinicians may prolong the polymerization time to increase their confidence in the procedure, while some clinicians reduce the duration of the polymerization to complete the restoration quickly. This in vitro study demonstrated that polymerization time did not change the microhardness and roughness of the bulk-fill composites before bleaching procedures. However, after bleaching, polymerization time altered the surface roughness and microhardness of the bulk-fill composites. From this point of view, it may be concluded that the polymerization time recommended by the manufacturers should be taken into consideration. In this way, the durability of the composite material in the oral environment might be positively affected.

## Figures and Tables

**Table 1:** Materials, manufacturer, and chemical composition.

Material	Abbreviation	Manufacturer	Composition
Filtek Bulk Fill nano-RBC	FB	3M ESPE, USA	AUDMA, UDMA, 1.12-dodecane-DMA, 4-11 nm zirconia particles, 20 nm silica and 100 nm ytterbium trifluoride fillers CQ, EDMAB (76.5% by weight)
SonicFill nanohybrid RBC	SF	Kerr, USA	Bis-GMA, TEGDMA, EBPDMA, SiO_2_, glass, oxide (83.5% by weight), CQ
Bite & white	BW	Cavex, Netherland	16% carbamide peroxide (equivalent to 6% hydrogen peroxide) 0.2% potassium nitrate desensitizer 0.1% sodium fluoride desensitizer, carbomer, glycerin, EDTA, potassium hydroxide, peppermint oil, water

Abbreviations: Bis-GMA, bisphenol-A diglycidyl ether dimethacrylate; EBPDMA, ethoxylated bisphenol-A-dimethacrylate; TEGDMA, triethylene glycol dimethacrylate; UDMA, urethane dimethacrylate.

**Table 2:** B/T microhardness ratio of the groups before bleaching, the standard deviations and the significant differences between the groups.

Time (s)	FB	SF
10	76.57 ± 11.67^Aa^	78.59 ± 7.95^Aa^
20	84.88 ± 4.40^Aa^	83.80 ± 9.17^Aa^
30	90.77 ± 5.02^Aa^	84.99 ± 3.66^Aa^

Different capital letters show the significant differences between the same columns (*p* < 0.05). Different small letters show the significant differences between the same lines in each group (*p* < 0.05).

**Table 3:** Microhardness measurements (VHN), the standard deviations of composites' top surfaces before and after bleaching and the significant difference between the groups.

Time (s)	Before bleaching			After bleaching			*p* values among before-after bleaching measurements	
	FB	SF	*p*	FB	SF	*p*	*p* (FB groups)	*p* (SF groups)
10	69.25 ± 8.26^Aa^	76.36 ± 3.49^Aa^	0.222	64.88 ± 5.13^Aa^	70.67 ± 6.96^Aa^	0.222	0.225	0.225
20	76.55 ± 5.63^Aa^	78.36 ± 5.57^Aa^	0.690	71.83 ± 6.63^Aab^	78.05 ± 1.66^Ba^	0.032	0.104	0.893
30	79.73 ± 2.36^Aa^	73.19 ± 3.80^Ba^	0.016	75.79 ± 2.28^Ab^	75.22 ± 7.26^Aa^	0.690	0.043	0.500
*p*	0.085	0.395		0.021	0.160			

Different capital letters show the significant differences between the same columns (*p* < 0.05). Different small letters show the significant differences between the same lines in each group (*p* < 0.05).

**Table 4:** Percentage alteration of microhardness values of composites (between before and after bleaching measurements) and *p* values.

Polymerization time	FB % alteration	SF % alteration	*p*
10 s	-5.7 ± 9.5^Aa^	-7.3 ± 10.5^Aa^	0.690
20 s	-6.1 ± 6.6^Aa^	0.4 ± 10.4^Aa^	0.548
30 s	-4.9 ± 4.2^Aa^	2.9 ± 10.5^Aa^	0.151
*p*	0.827	0.454	

Different capital letters show the significant differences between the same columns (*p* < 0.05). Different small letters show the significant differences between the same lines in each group (*p* < 0.05).

**Table 5:** Mean values, the standard deviation of surface roughness (Ra, *μ*m) measurements and the significant difference between the groups.

Time (s)	Before bleaching			After bleaching			*p* values among before-after bleaching measurements	
	FB	SF	*p*	FB	SF	*p*	*p* (FB)	*p* (SF)
10	0.51 ± 0.22^Aa^	0.49 ± 0.19^Ba^	0.868	0.51 ± 0.27^Ed^	0.62 ± 0.59^FGd^	0.668	0.997	0.494
20	0.67 ± 0.39^Ab^	0.64 ± 0.30^BCb∗^	0.820	1.03 ± 0.52^Ee^	1.39 ± 1.09^FHe∗^	0.183	0.072	<0.001
30	0.43 ± 0.26^Ac^	0.28 ± 0.19^BDc∗^	0.196	0.50 ± 0.27^Ef^	0.89 ± 0.48^Ff∗^	0.152	0.730	0.003
*p*	0.140	0.013		0.086	0.019			

Different capital letters show the significant differences between the same columns (*p* < 0.05). Different small letters show the significant differences between the same lines in each group (*p* < 0.05). ^∗^ signs show the significant differences between the before-after measurements in each material (*p* < 0.05).

**Table 6:** Percentage alteration of roughness values of composites (between before and after bleaching measurements) and *p* values.

Polymerization time	FB % alteration	SF % alteration	*p*
10 s	16.7 ± 74^Aa^	53.6 ± 155.2^BCa^	0.684
20 s	95.6 ± 158.3^Ab^	225.4 ± 448.6^Bb^	0.796
30 s	51 ± 114.5^Ac^	271 ± 210.8^BDd^	0.011
*p*	0.310	0.016	

Different capital letters show the significant differences between the columns (*p* < 0.05). Different small letters show the significant differences between the lines in each group (*p* < 0.05).

## Data Availability

The data used to support the findings of this study are included within the article.

## References

[1] Benetti A., Havndrup-Pedersen C., Honoré D., Pedersen M., Pallesen U. (2015). Bulk-fill resin composites: polymerization contraction, depth of cure, and gap formation. *Operative Dentistry*.

[2] Kim K.-H., Ong J. L., Okuno O. (2002). The effect of filler loading and morphology on the mechanical properties of contemporary composites. *The Journal of Prosthetic Dentistry*.

[3] Kawaguchi M., Fukushima T., Miyazaki K. (2016). The relationship between cure depth and transmission coefficient of visible-light-activated resin composites. *Journal of Dental Research*.

[4] Soh M. S., Yap A. U. J., Siow K. S. (2004). Comparative depths of cure among various curing light types and methods. *Operative Dentistry*.

[5] Hickey A., Lynch C. D., Ray N. J., Burke F. M., Hannigan A. (2002). Surface microhardness of a resin composite exposed to pulse-delayed plasma arc lamp irradiation, in vitro. *European journal of Prosthodontics and Restorative Dentistry*.

[6] Chung K. H., Greener E. H. (1990). Correlation between degree of conversion, filler concentration and mechanical properties of posterior composite resins. *Journal of Oral Rehabilitation*.

[7] Leprince J., Devaux J., Mullier T., Vreven J., Leloup G. (2010). Pulpal-temperature rise and polymerization efficiency of LED curing lights. *Operative Dentistry*.

[8] Castro F. L. A. d., Campos B. B., Bruno K. F., Reges R. V. (2013). Temperature and curing time affect composite sorption and solubility. *Journal of Applied Oral Science*.

[9] Rueggeberg F. A., Giannini M., Arrais C. A. G., Price R. B. T. (2017). Light curing in dentistry and clinical implications: a literature review. *Brazilian Oral Research*.

[10] Greenwall L., Greenwall L. (2001). *Bleaching Techniques in Restorative Dentistry : An Illustrated Guide*.

[11] Hannig C., Duong S., Becker K., Brunner E., Kahler E., Attin T. (2007). Effect of bleaching on subsurface micro-hardness of composite and a polyacid modified composite. *Dental Materials*.

[12] Turker S. B., Biskin T. (2002). The effect of bleaching agents on the microhardness of dental aesthetic restorative materials. *Journal of Oral Rehabilitation*.

[13] Li Q., Yu H., Wang Y. (2009). Colour and surface analysis of carbamide peroxide bleaching effects on the dental restorative materials in situ. *Journal of Dentistry*.

[14] Bailey S. J., Swift EJ Jr (1992). Effects of home bleaching products on composite resins. *Quintessence International*.

[15] Anusavice K. J., Shen C., Rawls H. R. (2013). Phillips’ science of dental materials. *Journal of Chemical Information and Modeling*.

[16] Steinberg D., Mor C., Dogan H., Zacks B., Rotstein I. (1999). Effect of salivary biofilm on the adherence of oral bacteria to bleached and non-bleached restorative material. *Dental Materials*.

[17] Kaya M. S., Bakkal M., Durmus A., Durmus Z. (2018). Structural and mechanical properties of a giomer-based bulk fill restorative in different curing conditions. *Journal of Applied Oral Science*.

[18] Fleming G. J. P., Awan M., Cooper P. R., Sloan A. J. (2008). The potential of a resin-composite to be cured to a 4mm depth. *Dental Materials*.

[19] Poggio C., Lombardini M., Gaviati S., Chiesa M. (2012). Evaluation of Vickers hardness and depth of cure of six composite resins photo-activated with different polymerization modes. *Journal of Conservative Dentistry*.

[20] Bouschlicher M. R., Rueggeberg F. A., Wilson B. M. (2004). Correlation of bottom-to-top surface microhardness and conversion ratios for a variety of resin composite compositions. *Operative Dentistry*.

[21] Knezević A., Tarle Z., Meniga A., Sutalo J., Pichler G., Ristić M. (2001). Degree of conversion and temperature rise during polymerization of composite resin samples with blue diodes. *Journal of Oral Rehabilitation*.

[22] Leprince J. G., Palin W. M., Hadis M. A., Devaux J., Leloup G. (2013). Progress in dimethacrylate-based dental composite technology and curing efficiency. *Dental Materials*.

[23] Leprince J. G., Palin W. M., Vanacker J., Sabbagh J., Devaux J., Leloup G. (2014). Physico-mechanical characteristics of commercially available bulk-fill composites. *Journal of Dentistry*.

[24] Aguiar F. H. B., Braceiro A. T. B., Ambrosano G. M. B., Lovadino J. R. (2005). Hardness and diametral tensile strength of a hybrid composite resin polymerized with different modes and immersed in ethanol or distilled water media. *Dental Materials*.

[25] Borges B. C. D., Bezerra G. V. G., Mesquita J. A. (2012). Filler morphology of resin-based low-viscosity materials and surface properties after several photoactivation times. *Acta Odontologica Scandinavica*.

[26] Lima A. F., de Andrade K. M. G., da Cruz Alves L. E. (2012). Influence of light source and extended time of curing on microhardness and degree of conversion of different regions of a nanofilled composite resin. *European Journal of Dentistry*.

[27] Halvorson R. H., Erickson R. L., Davidson C. L. (2002). Energy dependent polymerization of resin-based composite. *Dental Materials*.

[28] Malkondu Ö., Yurdagüven H., Say E., Kazazoğlu E., Soyman M. (2011). Effect of bleaching on microhardness of esthetic restorative materials. *Operative Dentistry*.

[29] Yu H., Li Q., Cheng H., Wang Y. (2011). The effects of temperature and bleaching gels on the properties of tooth-colored restorative materials. *The Journal of Prosthetic Dentistry*.

[30] Blackham J. T., Vandewalle K. S., Lien W. (2009). Properties of hybrid resin composite systems containing prepolymerized filler particles. *Operative Dentistry*.

[31] Polydorou O., Hellwig E., Auschill T. M. (2006). The effect of different bleaching agents on the surface texture of restorative materials. *Operative Dentistry*.

[32] Attin T., Hannig C., Wiegand A., Attin R. (2004). Effect of bleaching on restorative materials and restorations—a systematic review. *Dental Materials*.

[33] Wang L., Garcia F. C. P., de Araújo P., Franco E. B., Mondelli R. F. L. (2004). Wear resistance of packable resin composites after simulated toothbrushing test. *Journal of Esthetic and Restorative Dentistry*.

[34] Badra V. V., Faraoni J. J., Ramos R. P., Palma-Dibb R. G. (2005). Influence of different beverages on the microhardness and surface roughness of resin composites. *Operative Dentistry*.

[35] Yazici A. R., Tuncer D., Antonson S., Onen A., Kilinc E. (2010). Effects of delayed finishing/polishing on surface roughness, hardness and gloss of tooth-coloured restorative materials. *European Journal of Dentistry*.

[36] Mourouzis P., Koulaouzidou E. A., Helvatjoglu-Antoniades M. (2013). Effect of in-office bleaching agents on physical properties of dental composite resins. *Quintessence International*.

